# The Mechanism for Type I Interferon Induction by *Mycobacterium tuberculosis* is Bacterial Strain-Dependent

**DOI:** 10.1371/journal.ppat.1005809

**Published:** 2016-08-08

**Authors:** Kirsten E. Wiens, Joel D. Ernst

**Affiliations:** 1 Department of Pathology, New York University School of Medicine, New York, New York, United States of America; 2 Division of Infectious Disease, Department of Medicine, New York University School of Medicine, New York, New York, United States of America; 3 Department of Microbiology, New York University School of Medicine, New York, New York, United States of America; University of Washington, UNITED STATES

## Abstract

Type I interferons (including IFNαβ) are innate cytokines that may contribute to pathogenesis during *Mycobacterium tuberculosis* (Mtb) infection. To induce IFNβ, Mtb must gain access to the host cytosol and trigger stimulator of interferon genes (STING) signaling. A recently proposed model suggests that Mtb triggers STING signaling through bacterial DNA binding cyclic GMP-AMP synthase (cGAS) in the cytosol. The aim of this study was to test the generalizability of this model using phylogenetically distinct strains of the Mtb complex (MTBC). We infected bone marrow derived macrophages with strains from MTBC Lineages 2, 4 and 6. We found that the Lineage 6 strain induced less IFNβ, and that the Lineage 2 strain induced more IFNβ, than the Lineage 4 strain. The strains did not differ in their access to the host cytosol and IFNβ induction by each strain required both STING and cGAS. We also found that the three strains shed similar amounts of bacterial DNA. Interestingly, we found that the Lineage 6 strain was associated with less mitochondrial stress and less mitochondrial DNA (mtDNA) in the cytosol compared with the Lineage 4 strain. Treating macrophages with a mitochondria-specific antioxidant reduced cytosolic mtDNA and inhibited IFNβ induction by the Lineage 2 and 4 strains. We also found that the Lineage 2 strain did not induce more mitochondrial stress than the Lineage 4 strain, suggesting that additional pathways contribute to higher IFNβ induction. These results indicate that the mechanism for IFNβ by Mtb is more complex than the established model suggests. We show that mitochondrial dynamics and mtDNA contribute to IFNβ induction by Mtb. Moreover, we show that the contribution of mtDNA to the IFNβ response varies by MTBC strain and that additional mechanisms exist for Mtb to induce IFNβ.

## Author Summary

Bacterial strains from the *Mycobacterium tuberculosis* complex (MTBC) infect one in three humans, however not all infected individuals progress to active tuberculosis disease. It is unknown why immunocompetent individuals develop tuberculosis, and this presents a significant challenge in preventing the disease. One proposed explanation is that individuals that progress produce higher levels of the innate cytokine type I interferon, due to bacterial and/or host determinants. Therefore we set out to determine whether MTBC strains from distinct phylogenetic lineages induce distinct levels of type I interferon, and to determine a mechanism of type I interferon induction by each strain. We found that a Lineage 6 strain induced lower levels of type I interferon in macrophages than Lineage 2 and 4 strains. Additionally, this strain induced low levels of mitochondrial stress. We showed that improving mitochondrial function by treating macrophages with a mitochondria-specific antioxidant reduced type I interferon induction by Lineage 2 and 4 strains. Our results indicate that differential mitochondrial stress contributes to differential type I interferon induction by distinct MTBC strains. This suggests that mitochondria-specific antioxidants may be a means of reducing the pathogenic type I interferon response.

## Introduction

Type I interferons (including IFNαβ) are innate cytokines that are protective during most viral infections, but may be pathogenic during infections with bacteria such as *Mycobacterium tuberculosis* (Mtb) [1]. Studies have shown that active tuberculosis (TB) is associated with expression of interferon-inducible genes [2, 3], lepromatous *Mycobacterium leprae* lesions are enriched in IFNαβ-inducible mRNAs [4], and that interleukin-1 confers resistance to Mtb by limiting IFNαβ induction [5]. There is also evidence that IFNαβ is protective in certain contexts [6], and thus it is likely that a balance of this cytokine is required for optimal protection. Given the complex role of IFNαβ signaling during Mtb infection, discovering a model for how Mtb induces IFNβ gene transcription—the first step required for IFNαβ signaling—has been an active and challenging area of research.

Several groups have recently proposed a mechanism for IFNβ induction by Mtb. In this model, the first step in the pathway occurs when Mtb gains access to the host cytosol [7], such as through phagosome permeabilization [8]. The second step is initiation of the STING (stimulator of interferon genes) signaling pathway. STING can be triggered by bacterial cyclic dinucleotides [9] or through DNA binding to cGAS (cyclic GMP-AMP synthase) in the cytosol [10–12]. The established model suggests that bacterial DNA shed from cytosolic mycobacteria binds to and activates cGAS in order to induce IFNβ [12, 13]. However, this model does not take into account the contribution of DNA from other sources, such as mitochondria. Furthermore, this model has only been tested with strains from MTBC Lineage 4 and it is unknown whether the mechanism is generalizable to strains from other phylogenetic lineages.

The two important outstanding questions that we address in this study are: 1) Do phylogenetically distinct mycobacterial strains induce distinct levels of IFNβ *in vitro*? and 2) What are the mechanisms underlying IFNβ induction by these strains? Identifying a mechanism by which distinct MTBC strains promote IFNαβ induction would provide crucial insight into a mechanism of Mtb pathogenesis and into the evolution and diversity of mycobacterial strains.

Therefore, in order to examine the model for IFNβ induction by Mtb, we infected bone marrow derived macrophages (BMDM) with bacterial strains from three phylogenetically distinct MTBC lineages and assayed cytosolic signaling and IFNβ induction by each. The MTBC strains we chose were 1182 (Lineage 6; also known as *M*. *africanum*) [14, 15], H37Rv (Lineage 4) [16], and 4334 (Lineage 2) [17, 18] (S1 Table). We find that 1182/Lineage 6 induces significantly less, and 4334/Lineage 2 induces significantly more, IFNβ than H37Rv/Lineage 4. Additionally, we find that strain differences in IFNβ induction are not due to differences in cytosolic access or bacterial DNA shedding. Instead, we provide evidence that IFNβ differences are due, at least partially, to differences in mitochondrial stress and mitochondrial DNA (mtDNA) in the cytosol. Moreover, we show that additional mechanisms exist for Mtb to induce IFNβ and thus that the mechanism for IFNβ induction by Mtb is much more complex than the established model has implied.

## Results

### IFNβ induction is bacterial strain-dependent during Mtb infection

**Fig 1:**
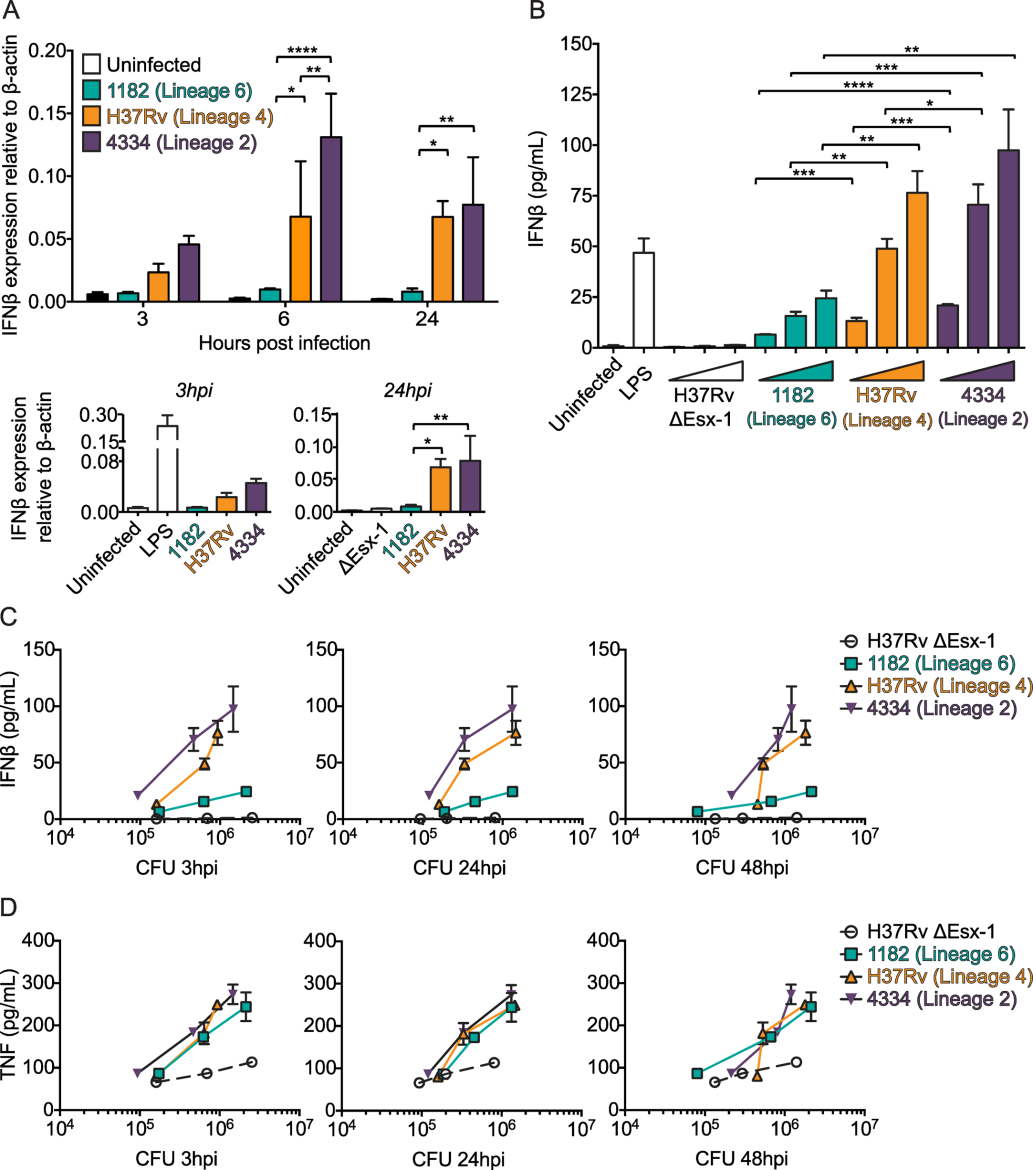
IFNβ induction is bacterial strain-dependent during Mtb infection. A) BMDM were infected with the indicated mycobacterial strains at an MOI of 5 or stimulated with LPS (10 ng/mL). RNA was harvested for IFNβ mRNA quantification by qRT-PCR at 3, 6, and 24 hr post infection. *p<0.05, **p<0.01, ****p<0.0001 by two-way repeated measures ANOVA with Tukey post-tests; means ± SD (n = 3). B) BMDM were infected with the indicated bacterial strains at MOI of 1, 5, and 10 or stimulated with LPS (10 ng/mL). Supernatants were collected for IFNβ protein quantification by ELISA at 48 hr post infection. *p<0.05, **p<0.01, ***p<0.001, ****p<0.0001 by one-way ANOVA with Tukey post-tests for each MOI; means ± SD (n = 3). Results are representative of 3 independent experiments. C-D) Cell lysates from the experiment shown in Fig 1B were collected at 3, 24, and 48 hr post infection. Colony forming units (CFU) were quantified by serial dilution on 7H11 agar plates. CFU for each MOI at each time point are plotted on the x-axes and the corresponding IFNβ (C) and TNF (D) secretion at 48 hr post infection is plotted on the y-axes.

To determine whether distinct mycobacterial strains induce distinct levels of IFNβ *in vitro*, we infected bone marrow derived macrophages (BMDM) with strains from three distinct phylogenetic lineages (S1 Table). We found that 1182/Lineage 6 induced less IFNβ, and 4334/Lineage 2 induced more IFNβ, than H37Rv/Lineage 4 (Fig 1). Differences in mRNA expression were detected at 3 hr post infection and continued at 6 and 24 hr post infection (Fig 1A). Differences in secreted protein were detected at 48 hr post infection and were consistent across several multiplicities of infection (MOI) (Fig 1B). The H37Rv ΔEsx-1 strain that is unable to access the cytosol [8, 19] did not induce IFNβ (Fig 1A and 1B).

Importantly, the differences in IFNβ protein secretion were not due to differences in bacterial numbers or intracellular survival (Fig 1C), and were not mirrored by differences in tumor necrosis factor (TNF) secretion (Fig 1D). To verify this, we included colony forming units (CFU) and TNF as covariates in an ANCOVA model (S2 Table). We found that CFU and TNF did not explain variation in IFNβ, while bacterial strain did, at each MOI. This suggested that IFNβ induction by Mtb was bacterial strain-dependent and that strain differences were not explained by differences in bacterial growth or accompanied by global differences in inflammatory cytokine production. We also assayed interleukin-1 (IL-1) secretion, which negatively regulates IFNβ [5]. We found that IL-1 levels were below the limit of detection during infection with each strain, and thus variation in IL-1 likely did not explain IFNβ variation in this system. Therefore we hypothesized that differences in IFNβ induction were due to differences in cytosolic signaling.

### Access to the host cytosol does not vary by mycobacterial strain

**Fig 2:**
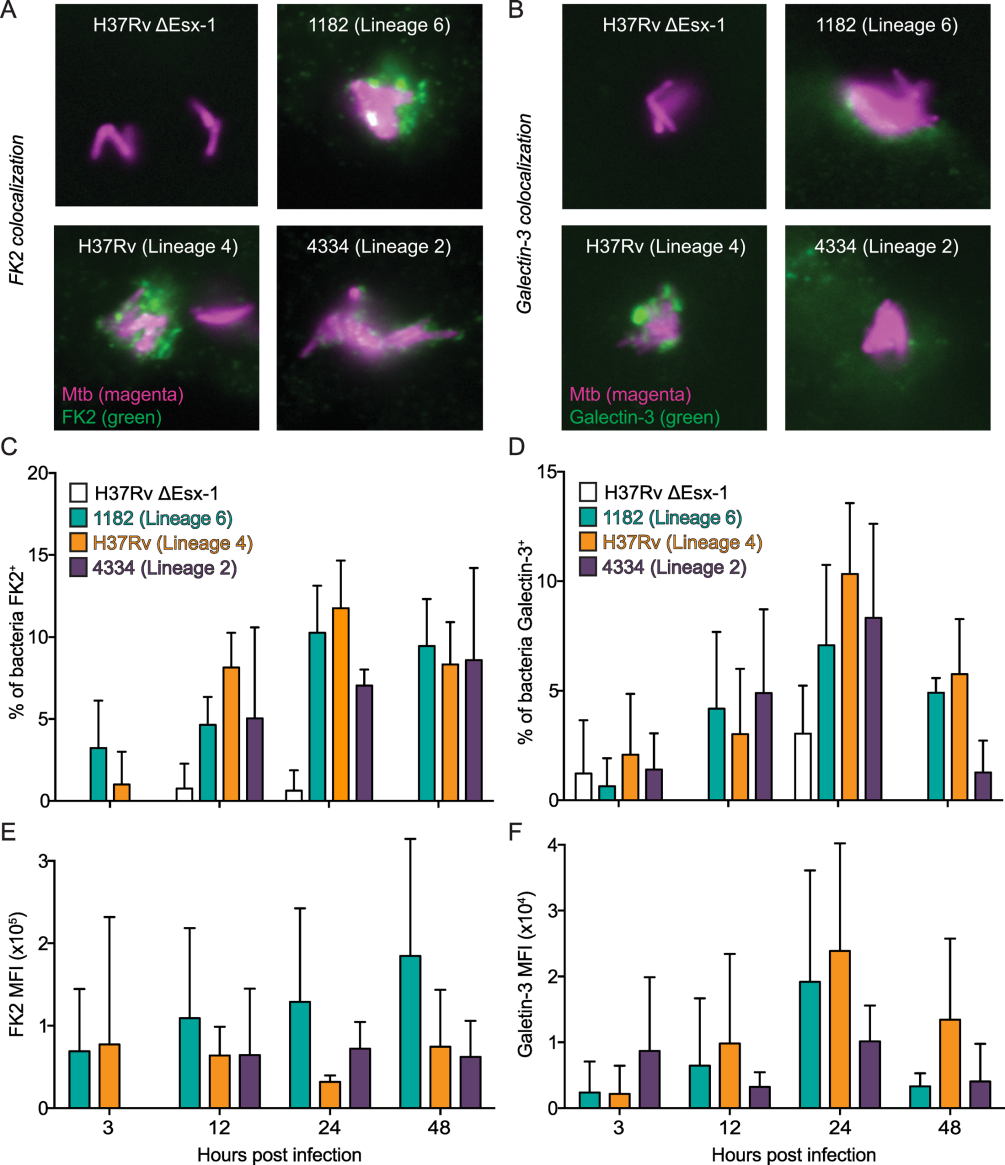
Access to the host cytosol does not vary by mycobacterial strain. A-B) BMDM were infected with the indicated dsRed-expressing mycobacterial strains at an MOI of 1. BMDM were fixed overnight and stained for FK2 and Galectin-3 at 3, 12, 24, and 48 hr post infection. Representative images are shown for FK2 colocalization at 48 hr post infection (A) and Galectin-3 colocalization at 24 hr post infection (B), with bacteria shown in magenta and FK2 and Galectin-3 shown in green. C-D) 10 images were captured at 100x magnification for each chamber well and the percent of bacteria that colocalized with FK2 (C) and Galectin-3 (D) was calculated for each strain. E-F) Mean fluorescence intensity (MFI) of the FK2 (E) and Galectin-3 (F) staining directly surrounding each colocalized bacterium was calculated using ImageJ. Results are representative of 1–3 independent experiments. All differences in percent colocalization and MFI between 1182, H37Rv, and 4334 were not significant (p>0.05) by two-way repeated measures ANOVA with Tukey post-tests; means ± SD (n = 4).

To induce IFNβ gene transcription, Mtb must gain access to the host cytosol [7]. To determine whether cytosolic access varied by mycobacterial strain, we infected BMDM and assayed colocalization of each strain with FK2 and Galectin-3. FK2 labels ubiquitinated proteins and thus labels bacteria that gain access to the cytosol and are ubiquitinated [20]. Galectin-3 labels damaged vacuole membranes [21]. There were no detectable differences in the percent of bacteria that colocalized with FK2 (Fig 2A and 2C) or Galectin-3 (Fig 2B and 2D) at any time point examined during infection with 1182/Lineage 6, H37Rv/Lineage 4, or 4334/Lineage 2. 4334/Lineage 2 interacted differently with FK2 and Galectin-3 than 1182/Lineage 6 and H37Rv/Lineage 4, as indicated by staining patterns (Fig 2A and 2B), however this was not associated with IFNβ induction. In addition, we measured the mean fluorescence intensity (MFI) of FK2 and Galectin-3 surrounding each colocalized bacterium and found no significant differences between the strains (Fig 2E and 2F). The ΔEsx-1 negative control strain did not colocalize with FK2 or Galectin-3. These data suggested that the differences in IFNβ induction between MTBC strains were not attributable to differences in access of the bacteria to the cytosol.

We confirmed these results using a LiveBLAzer FRET assay [8]. In this assay BMDM are incubated with Cephalosporin Coumarin Fluorescein 4 (CCF4), a cephalosporin substrate labeled with two fluorophores that form a fluorescent resonance energy transfer (FRET) pair. In BMDM where Mtb is sequestered in a phagosome, CCF4 is uncleaved and when excited at 409 nm emits fluorescence with a peak at 520 nm. In BMDM where Mtb gains access to the cytosol, Mtb’s endogenous β-lactamase cleaves CCF4 and disrupts the FRET signal and phagosome permeabilization is detected by BMDM that emit a signal at 450 nm. We found no difference in 450:520 nm fluorescence ratios between the three MTBC strains (S1 Fig), which confirmed the FK2 and Galectin-3 results. Together, these data indicated that differences in IFNβ induction between the MTBC strains were not due to differences in access to the host cytosol.

### IFNβ induction by each MTBC strain is dependent on STING and cGAS

**Fig 3:**
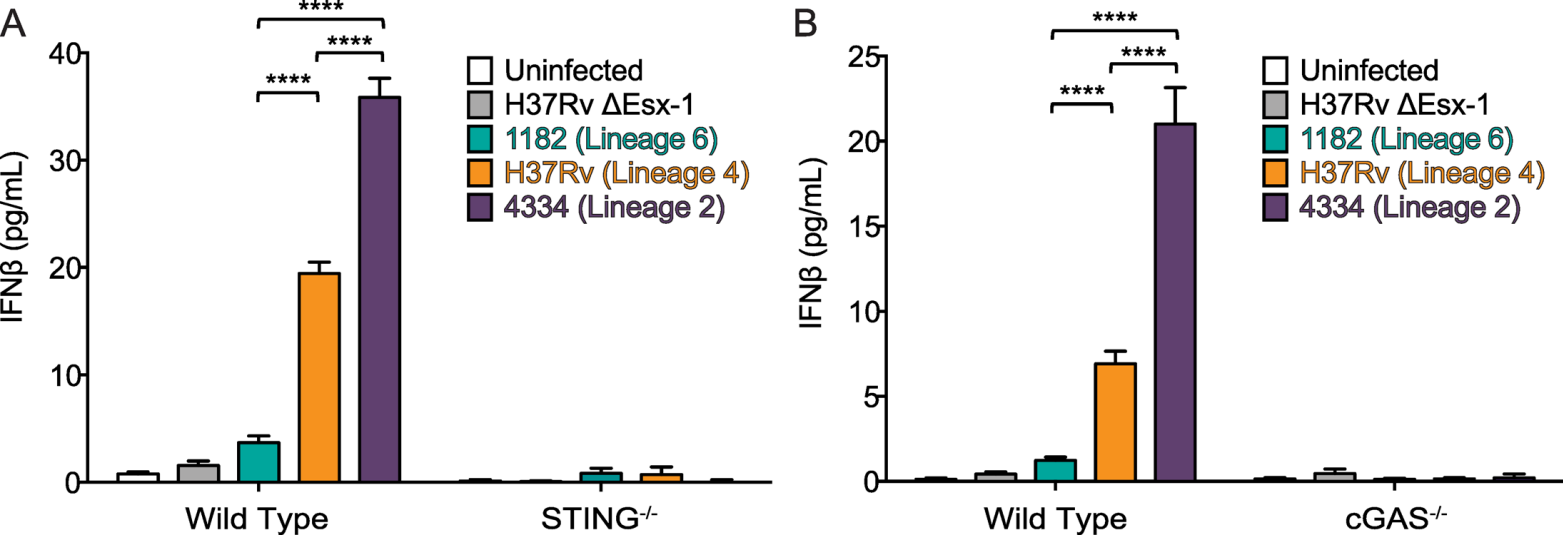
IFNβ induction by each MTBC strain is dependent on STING and cGAS. A) Wild type and STING^-/-^ BMDM were infected with the indicated mycobacterial strains at an MOI of 5. Supernatants were collected for IFNβ protein quantification by ELISA at 48 hr post infection. B) Wild type and cGAS^-/-^ BMDM were infected with the indicated bacterial strains at an MOI of 5. Supernatants were collected for IFNβ protein quantification by ELISA at 48 hr post infection. ****p<0.0001 by two-way ANOVA with Tukey post-tests; means ± SD (n = 3).

Once Mtb gains access to the cytosol it triggers STING signaling, either by bacterial cyclic dinucleotides [9] or through dsDNA binding to and activating cGAS in the cytosol [10–12]. To determine whether IFNβ induction by each strain was dependent on STING signaling we infected STING^-/-^ and wild type BMDM and assayed IFNβ protein secretion. We found that IFNβ induction by each strain was completely abrogated in the absence of STING (Fig 3A), indicating that IFNβ induction was STING-dependent, and suggested that under these conditions differential production of cGAMP or release of DNA into the cytosol may contribute to differential IFNβ induction.

Next we determined whether IFNβ induction by each strain was due to direct STING signaling or due to STING signaling via cGAS. Therefore we infected cGAS^-/-^ and wild type BMDM and assayed IFNβ protein secretion. We found that IFNβ induction by each strain was reduced in the absence of cGAS to the same degree that IFNβ induction was reduced in the absence of STING (Fig 3B). These data suggested that differences in IFNβ induction between the MTBC strains might depend on differences in the availability of cytosolic DNA for binding to and activating cGAS.

### Release of host, but not bacterial, DNA into the cytosol is bacterial strain-dependent during Mtb infection

**Fig 4:**
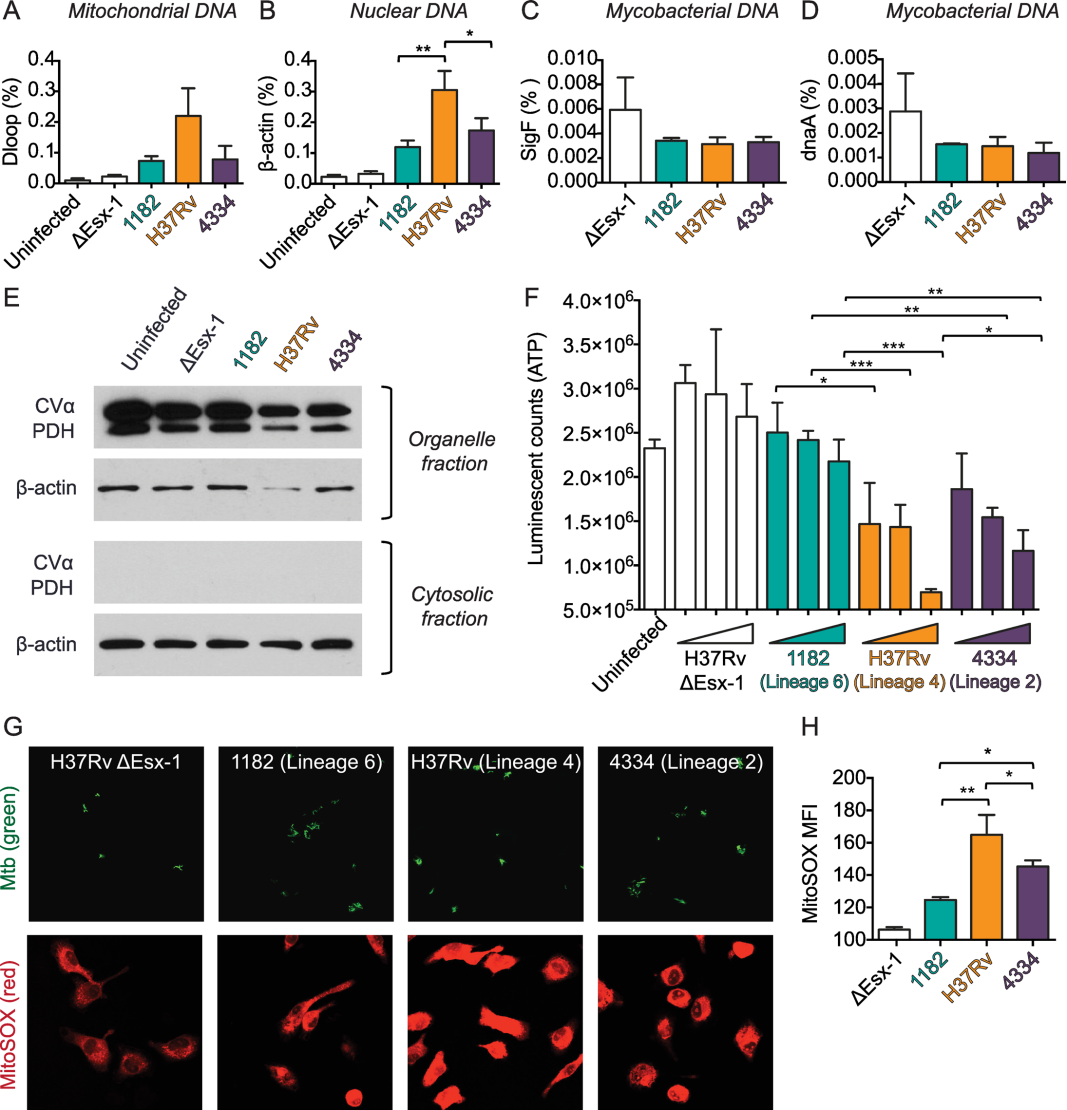
Release of host, but not bacterial, DNA into the cytosol is mycobacterial strain-dependent and is associated with mitochondrial stress. A-D) BMDM were infected with the indicated mycobacterial strains at an MOI of 5. 24 hr post infection, cells were collected and fractionated. Mitochondrial (A), nuclear (B), and bacterial (C,D) DNA in cytosolic fractions was quantified using gene-specific primers and normalized to the amount of each gene in lysates of unfractionated cells (shown as %). *p<0.05, **p<0.01 by one-way ANOVA with Tukey post-tests; means ± SD (n = 3). E) Mitochondrial proteins (CVα and PDH) in the organelle and cytosolic fractions were quantified on immunoblots. β-actin was used as the cytosolic loading control. Results are representative of 2 independent experiments. F) BMDM were cultured in the presence of galactose and absence of glucose for 24 hr and then infected with the indicated mycobacterial strains at MOI of 1, 5, and 10. 24 hr post infection luciferase and luciferin were added to cell lysates and ATP production was measured by luminescence. *p<0.05, **p<0.01, ***p<0.001 by one-way ANOVA with Tukey post-tests for each MOI; means ± SD (n = 3). G-H) BMDM were infected with the indicated GFP-expressing mycobacterial strains at an MOI of 5. Live BMDM were stained with MitoSOX at 24 hr post infection and then fixed overnight. G) Representative images are shown, with Mtb shown in green and MitoSOX shown in red. H) 5 pictures were taken at 60x magnification for each chamber well and mean fluorescence intensity (MFI) of MitoSOX was determined for each infected BMDM using ImageJ. Results are representative of 3 independent experiments. *p<0.05, **p<0.01 by one-way ANOVA with Tukey post-tests; means ± SD (n = 3).

cGAS is a cytosolic DNA sensor that can recognize dsDNA of microbial, nuclear or mitochondrial origin [22, 23]. To determine whether accumulation of dsDNA in the cytosol was mycobacterial strain-dependent we infected BMDM with each MTBC strain and quantified cytosolic DNA following cell fractionation. We found that infection with H37Rv/Lineage 4 was associated with increased mitochondrial and nuclear DNA in the cytosol compared with 1182/Lineage 6 (Fig 4A and 4B), and we found no differences in release of bacterial DNA (Fig 4C and 4D). The procedure for preparing subcellular fractions to quantitate DNA permeabilized phagosome membranes as well as the plasma membrane, as is indicated by bacterial DNA detected in the cytosolic fraction of cells infected with the ΔEsx-1 mutant (Fig 4C and 4D). To verify that the fractionation buffer did not permeabilize other organelle membranes, we used immunoblotting to determine that the mitochondrial proteins Complex Vα (CVα) and pyruvate dehydrogenase E1α (PDH) remained in the organelle fractions and were not found in the cytosol fractions (Fig 4E).

Given these data we hypothesized that reduced IFNβ induction by 1182/Lineage 6 was due to reduced mtDNA in the cytosol. Although we also found reduced nuclear DNA in the cytosol of 1182/Lineage 6-infected cells, a recent study has shown that mtDNA–and not nuclear DNA–stimulates IFNβ induction [24]. We further hypothesized that the reduced mtDNA in the cytosol was due to reduced mitochondrial stress in cells infected with 1182/Lineage 6.

### Release of host DNA into the cytosol during Mtb infection is associated with mitochondrial stress

MtDNA may accumulate in the cytosol under conditions of stress [25–27], and mitochondrial stress and mtDNA have been implicated in IFNβ induction [24, 28–31]. To determine whether the release of mtDNA into the cytosol was associated with mitochondrial stress, we infected BMDM with each Mtb strain and quantified ATP as a measure of the bioenergetic state of the cell. We infected BMDM cultured in media lacking glucose and supplemented with galactose to prevent BMDM from using glycolysis for ATP production. We found that 1182/Lineage 6 was associated with higher cellular ATP concentrations compared with H37Rv/Lineage 4, with ATP levels similar to uninfected cells and the negative control ΔEsx-1 (Fig 4F). This suggested that 1182/Lineage 6 infection might induce especially low levels of mitochondrial stress. These data were consistent with the finding of lesser quantities of mtDNA in the cytosol of BMDM infected with 1182/Lineage 6 (Fig 4A).

To further examine mitochondrial stress, we quantified superoxide production in BMDM following infection with each bacterial strain. Superoxide is a reactive oxygen species (ROS) that is a byproduct of mitochondrial oxidative phosphorylation as electrons that leak from the electron transport chain are transferred to molecular oxygen. Conditions of stress result in an increase in the number of leaking electrons and result in accumulation of superoxide [32, 33]. We found reduced mitochondrial superoxide production during 1182/Lineage 6 infection compared to H37Rv/Lineage 4 (Fig 4G and 4H). This supported our conclusion that 1182/Lineage 6 was associated with reduced mitochondrial stress.

Importantly, we also found that 4334/Lineage 2 was associated with less host DNA in the cytosol (Fig 4A and 4B) and less mitochondrial stress (Fig 4F and 4G) than H37Rv/Lineage 4. This indicated that 4334/Lineage 2 induced high IFNβ levels by a different, additional mechanism.

### Mitochondrial stress and cytosolic mtDNA contribute to IFNβ induction by MTBC strains

**Fig 5:**
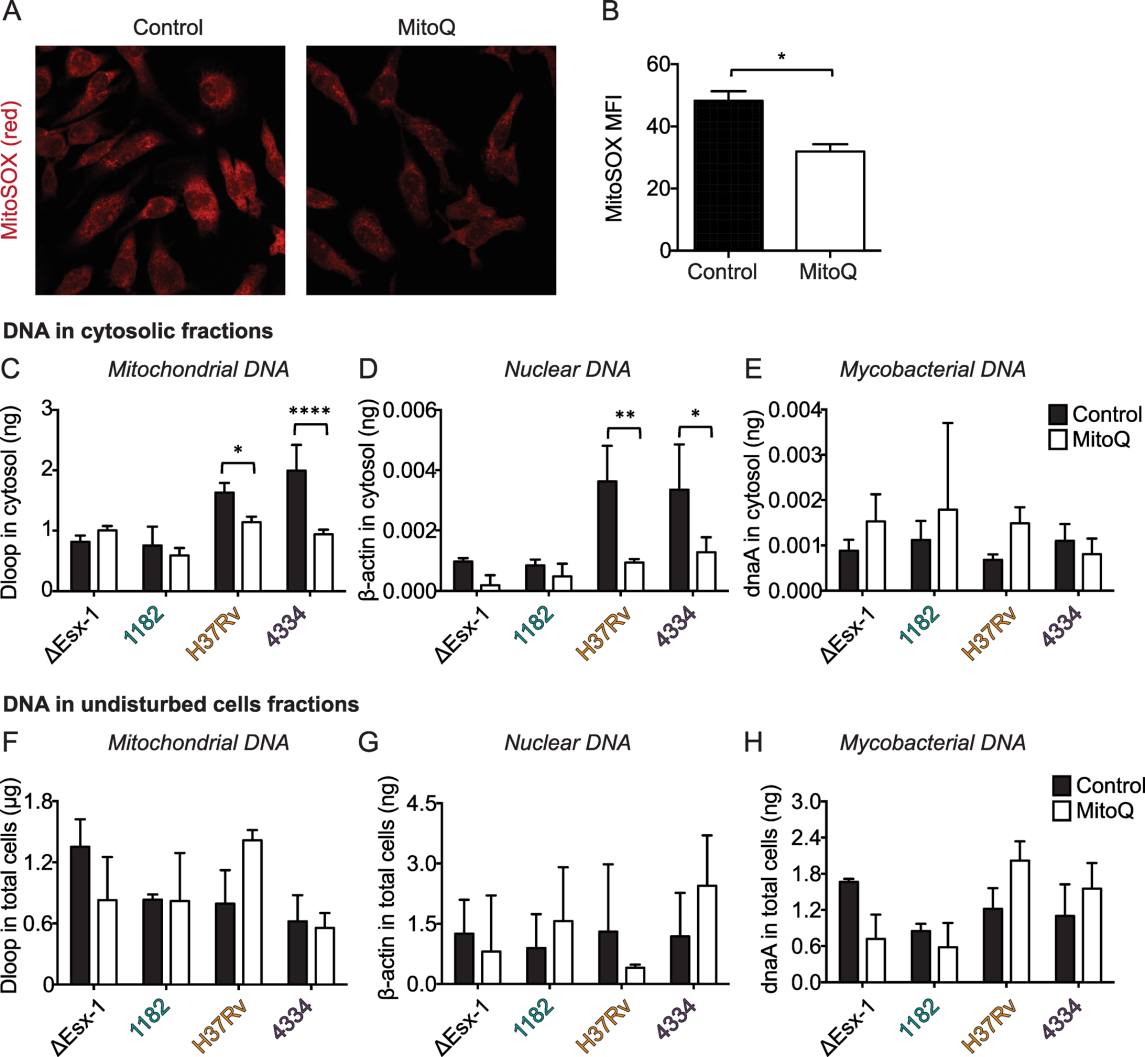
Mitochondrial stress contributes to accumulation of mtDNA in the cytosol during H37Rv/Lineage 4 and 4334/Lineage 2 infections. A-H) BMDM were treated with MitoQ or control (dTPP) for 4 hours and then infected with the indicated mycobacterial strains at an MOI of 5. A) Uninfected cells were stained with MitoSOX at the time of infection. B) Mean fluorescence intensity (MFI) of MitoSOX was determined using ImageJ. C-H) 24 hr post infection, cells were collected and fractionated. Amount of DNA in cytosolic (C-E) and undisturbed cell (F-H) fractions was determined using gene-specific primers as in Fig 4A–4D; amount in ng or μg was determined using standards that were generated independently of experimental samples and that contained abundant levels of each gene. *p<0.05, **p<0.01, ****p<0.0001 by two-way ANOVA with Sidak post-tests; means ± SD (n = 3).

Several studies have shown that mitochondrial ROS-induced oxidation of DNA contributes to the inflammatory response to DNA [25, 30, 34, 35]. Our data suggest that this may also be true for the IFNβ response to mycobacterial infection (Figs 1 and 4). To determine whether mycobacterial strain-dependent differences in IFNβ were due to differential accumulation of ROS, we treated BMDM with the antioxidant MitoQ and assayed accumulation of cytosolic DNA and IFNβ protein secretion induced by each strain. MitoQ is coenzyme Q attached to a lipophilic triphenylphosphonium cation. Coenzyme Q is a strong reducing agent and thus acts as an antioxidant by transferring electrons to superoxide. The lipophilic cation causes accumulation of the molecule specifically in the mitochondria [36]. We used decyltriphenylphosphonium bromide (dTPP) as the negative control. We found that MitoQ treatment partially reduced superoxide accumulation (Fig 5A and 5B). Higher doses of MitoQ were toxic to the cells and therefore we were not able to completely eliminate superoxide accumulation. Correspondingly, we found that MitoQ treatment reduced mtDNA accumulation in the cytosol during H37Rv/Lineage 4 and 4334/Lineage 2 infections (Fig 5C), but did not impact accumulation of bacterial DNA (Fig 5E). MitoQ treatment also reduced nuclear DNA accumulation in the cytosol, but the levels of nuclear DNA in the cytosol were lower than the levels of mtDNA (Fig 5D). MitoQ treatment had no significant effect on total cellular levels of mitochondrial, nuclear, or bacterial DNA (Fig 5F–5H).

**Fig 6:**
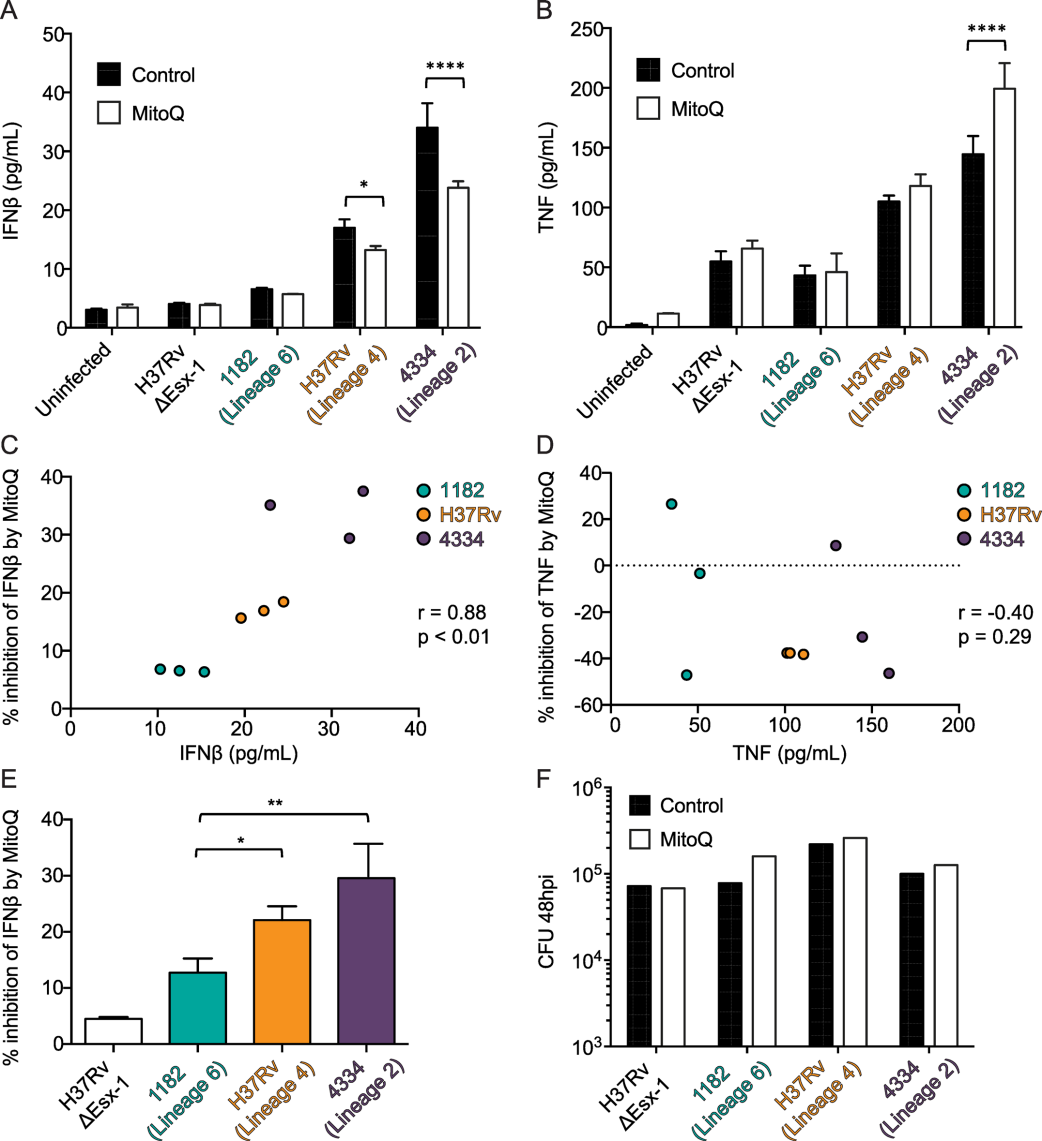
Mitochondrial stress contributes to IFNβ induction by MTBC strains. A-F) BMDM were treated with MitoQ or control (dTPP) for 4 hours and then infected with the indicated bacterial strains at an MOI of 5. A-B) 48 hr post infection supernatants were collected for IFNβ (A) and TNF (B) protein quantification by ELISA. *p<0.05, ****p<0.0001 by two-way ANOVA with Sidak post-tests; means ± SD (n = 3). IFNβ results are representative of 3 independent experiments. TNF comparison between control and MitoQ treatment is representative of 3 independent experiments, but the differences in TNF between Mtb strains varied between experiments. C-D) Percent inhibition of IFNβ (C) or TNF (D) induction during MitoQ treatment was calculated for each replicate (n = 3) during infection with each strain. Pearson correlation coefficient (r) of percent inhibition (y-axis) and cytokine secretion (x-axis) is shown. G) Percent inhibition of IFNβ during MitoQ treatment. *p<0.05, **p<0.01 by one-way ANOVA with Tukey post-tests; means ± SD (n = 3). F) Lysates from replicates (n = 3) of the experiment shown in Fig 6A–6E were pooled and CFU were quantified by serial dilution on 7H11 agar plates.

Furthermore, we found that MitoQ treatment partially reduced IFNβ induction by H37Rv/Lineage 4 and 4334/Lineage 2 (Fig 6A), and that the percent of IFNβ inhibited by MitoQ treatment was positively correlated with the amount of IFNβ a strain induced (Pearson r = 0.88, p = 0.002 and Fig 6C and 6E). IFNβ reduction during MitoQ treatment was not mirrored by a reduction in TNF (Fig 6B and 6D) or by a reduction in CFU (Fig 6F). In addition, the amount of mtDNA in the cytosol of infected cells positively correlated with IFNβ induction (Pearson r = 0.73, p = 0.0006 and S2 Fig), while bacterial DNA did not (Pearson r = -0.23, p = 0.36 and S2 Fig). Nuclear DNA in the cytosol also correlated with IFNβ induction, but the correlation was not a strong as for mtDNA (Pearson r = 0.60, p = 0.01 and S2 Fig). These data indicated that IFNβ induction by the MTBC strains was due, at least in part, to mitochondrial stress and cytosolic mtDNA accumulation.

## Discussion

**Fig 7:**
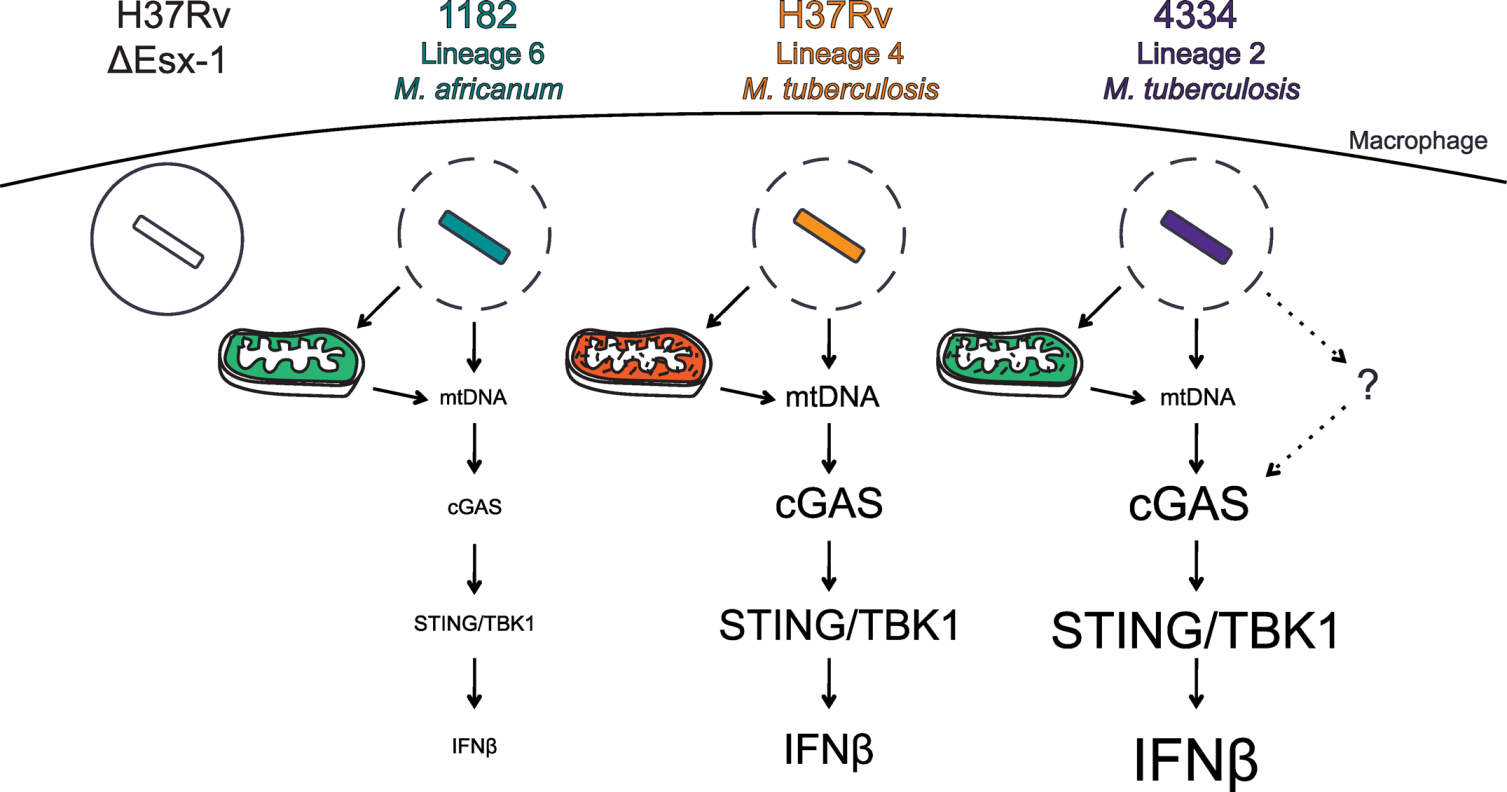
Model for mycobacterial strain-dependent IFNβ induction. We propose a model in which mitochondrial stress and mtDNA contribute to IFNβ induction by Mtb. 1182/Lineage 6 accesses the host cytosol to the same extent as H37Rv/Lineage 4 and 4334/Lineage 2. However, this strain induces less mitochondrial stress and less accumulation of mtDNA in the cytosol, which contributes to lower IFNβ induction. H37Rv/Lineage 4 induces more mitochondrial stress and more accumulation of mtDNA in the cytosol, which contributes to increased IFNβ induction. Our data suggest that 4334, and perhaps other strains, provide an additional cGAS signal that has not yet been discovered.

In this study we show that the *M*. *tuberculosis* complex strain 1182 from Lineage 6 induces less mitochondrial ROS, less mtDNA in the cytosol, and lower IFNβ induction than H37Rv/Lineage 4. Further, we show that reducing mitochondrial ROS during Mtb infection reduces IFNβ induction. Therefore we propose that mitochondrial stress contributes to IFNβ induction by Mtb (Fig 7). We also show that 4334/Lineage 2 induces similar to lower levels of mitochondrial ROS and cytosolic mtDNA than H37Rv/Lineage 4, yet induces higher IFNβ induction. Thus we propose that 4334/Lineage 2 induces additional, unidentified pathways to promote IFNβ induction (Fig 7). Together these results show that the mechanism for IFNβ induction by Mtb is much more complex than the established model suggests.

Mitochondria are integral centers in the cell that recognize and emit danger signals–such as mtDNA and ROS–to direct cellular responses [37], including various innate immune responses [32]. Here we have shown that mitochondrial dynamics contribute to the IFNβ response to Mtb infection. This has already been shown in other pathological contexts. MtDNA instability and accumulation of mtDNA in the cytosol contribute to the cGAS-mediated IFNβ response to HSV-1 [28]. Additionally, systemic lupus erythematosus is associated with mitochondrial dysregulation and type I interferon signatures [38–40], oxidized mtDNA released from lupus neutrophils drives type I interferon [24], and mitochondrial antioxidants attenuate IFNβ responses and lupus-like disease in mice [30]. Our finding that mitochondrial stress contributes to IFNβ induction by distinct MTBC strains suggests that it will be worthwhile to determine how widespread this pathway may be in other infections or diseases.

An important remaining question is how 1182/Lineage 6 induces less mitochondrial stress than H37Rv/Lineage 4. It is possible that 1182/Lineage 6 secretes reduced levels of a virulence factor that targets and disrupts mitochondria. Examples of such virulence factors in other pathogens include *Staphylococcus aureus* α-toxin and *Streptococcus pneumoniae* pneumolysin [41]. 1182/Lineage 6 contains a premature stop codon in Rv3879c of the Esx-1 gene locus [42], which does not impact ESAT-6 secretion [14] but may impact the secretion of other virulence factors. It is also possible that 1182/Lineage 6 secretes higher levels of an antioxidant that prevents mitochondrial toxicity. A third possibility is that 1182/Lineage 6 secretes reduced levels of a metabolite that feeds into the citric acid cycle and drives mitochondrial ROS. Succinate secretion is upregulated in mycobacteria under conditions of hypoxia [43] and perhaps a similar metabolic change occurs upon macrophage entry that drives mitochondrial ROS. This function been proposed for citrate produced by Salmonella during infection [44]. These possibilities are not mutually exclusive and studies are ongoing to address these questions.

Our results provide an alternative view of cytosolic signaling and IFNβ induction by Mtb than previous studies. First, we show that IFNβ induction is not necessarily a measure of cytosolic access, as 1182/Lineage 6 induced lower levels of IFNβ than the other two bacterial strains but, as indicated by three distinct assays, gained similar access to the host cytosol. Second, we show that bacterial DNA is probably not the predominant danger signal in the cytosol during Mtb infection; mtDNA released from stressed mitochondria likely binds cGAS to induce IFNβ. We did not observe complete reduction in IFNβ with MitoQ treatment. This could be due to residual superoxide and cytosolic mtDNA present in MitoQ-treated cells or it could be due to an additional mechanism that acts in concert with mitochondrial stress to promote IFNβ signaling. Perhaps bacterial DNA contributes to differential IFNβ induction but cannot be detected by the gene-specific primers that we used in our assays. We propose that we were able to discover this mechanism, while previous studies did not, for several reasons. First, we used diverse clinical isolates to test the model. Second, we used low bacterial doses (MOI of 1, 5, or 10) throughout the study and carefully controlled for any effect that CFU might have had on our results (Figs 1 and 6 and S2 Table). The study that showed mycobacterial DNA bound to cGAS used a high dose of bacteria (MOI of 20) and did not show CFU or mtDNA results [12].

Our results are consistent with previous studies that showed that Lineage 2 strains induce more IFNβ than Lineage 4 strains [45, 46]. This suggests that the ability to induce mitochondrial stress and/or IFNβ may contribute to the global dissemination and virulence of this lineage [47]. However, further studies would be required to determine how widespread this pathway is in Lineage 2. Our data also suggest that additional mechanisms exist for Lineage 2 strains to induce high levels of IFNβ, as 4334/Lineage 2 induced less mitochondrial stress than H37Rv/Lineage 4. We propose that 4334/Lineage 2 induces cGAS signaling by an as of yet undetermined mechanism. We noted that 4334/Lineage 2 interacted differently with FK2 and Galectin-3 than H37Rv/Lineage 4 and 1182/Lineage 6, indicating that this strain may behave differently in the host cytosol and may induce entirely distinct signaling pathways.

It also remains unknown if the reduced ability to induce mitochondrial stress and IFNβ is widespread in Lineage 6 strains. If so, these factors likely played a role in the evolution of Lineage 6. Lineage 6 strains exhibit low virulence and are geographically restricted to West Africa [48]; reduced ability to induce mitochondrial stress and IFNβ may contribute to this attenuation. Interestingly, Lineage 6 is currently not being outcompeted by Lineage 4 and Lineage 2 in areas where it is prevalent [49, 50]. Thus reduced mitochondrial toxicity and the ability to cause slower disease progression may have been selected for in Lineage 6, and future studies are warranted to investigate this possibility.

Ultimately, understanding IFNβ induction by Mtb may facilitate the development of host-directed TB treatments. Treating mice with prostaglandin E2 and zileuton, which limit IFNβ induction, confers tolerance to Mtb [5] and zileuton has been developed as a drug that can be administered to TB patients to enhance the efficacy of antibiotic treatment [51]. Our data suggest that mitochondria-specific antioxidants could be another means to limit the pathogenic type I interferon response. Studies that identify additional mechanisms by which Mtb induces type I interferon would then be indispensible as they could provide additional targets for TB prevention and therapy.

## Materials and Methods

### Ethics statement

All animal experiments were done in accordance with procedures approved by the NYU School of Medicine Institutional Animal Care and Use Committee (IACUC, Laboratory Animal Care Protocol: 150502–01). These IACUC regulations conformed to the national guidelines provided by the Guide for the Care and Use of Laboratory Animals of the National Institutes of Health.

### Cell cultures

BMDM were generated from 8–12 week old wild-type C57BL/6 mice from The Jackson Laboratory. BMDM were generated using 20% L929-cell-conditioned media for 7 days, unless otherwise indicated. For antioxidant treatment, BMDM were treated with 0.25 μM MitoQ (gift from Michael Murphy, Ph.D., MRC Mitochondrial Biology Unit, Cambridge, United Kingdom) or 0.25 μM decyltriphenylphosphonium bromide (Santa Cruz Biotechnology) for 4 hours prior to infection and throughout infection.

### Bacterial strains and culture conditions

H37Rv/Lineage 4 was obtained from American Type Culture Collection, 1182/Lineage 6 was obtained from an HIV-uninfected male with pulmonary tuberculosis in The Gambia (courtesy of Bouke de Jong, M.D., Ph.D., Institute of Tropical Medicine, Antwerp, Belgium), and 4334/Lineage 2 was obtained from a patient of unspecified HIV status with pulmonary tuberculosis in San Francisco (courtesy of Diane Ordway, Ph.D., Colorado State University, Fort Collins, USA and Midori Kato-Maeda, M.D., San Francisco General Hospital). Strains were grown at 37°C in Middlebrook 7H9 liquid or 7H11 solid media supplemented with 10% albumin, dextrose and catalase.

### Bacterial infections

Bacteria were grown to exponential phase, resuspended in 0.5% PBS-Tween 80, and centrifuged at 150 g for 8 min to remove clumped and dead bacteria. OD_580_:CFU ratios were calculated individually for each strain. Bacteria were added to BMDM at the MOI reported in the figure legend. Plates were incubated at 37°C under 5% CO_2_. Cells were washed 3–4 hr post infection and given fresh BMDM media.

### Cell fractionation

BMDM were infected in 6-well plates. Digitonin extracts were generated as previously described [52], with the digitonin concentration optimized for our assays. 24 hr post infection, cells were collected and resuspended in ice cold PBS. One eighth (by volume) of the suspension was subjected to bead-beating to lyse BMDM and bacteria and saved as the undisturbed cell normalization control. The remainder of the cells were resuspended in ice cold lysis buffer containing 150 mM NaCl, 50 mM HEPES pH 7.4, and 0.01% digitonin (Sigma). The homogenates were incubated end over end for 10 min at room temperature to allow selective membrane permeabilization and then centrifuged at 650 g for 5 min at 4°C to pellet intact cells. Supernatants were transferred to fresh tubes and centrifuged at maximum speed (20,800 g) for 10 min at 4°C to pellet organelles and obtain cytosolic supernatants. Organelle fractions were washed in PBS and resuspended in TN1 lysis buffer (50 mM Tris pH 8, 150 mM NaCl, 10% glycerol, 1 mM EDTA, 0.1% Triton-X, protease inhibitor cocktail). Organelle and cytosolic fractions were filtered with 0.22 μm SpinX filters (Corning). DNA and protein were extracted from undisturbed cell and cytosolic fractions by phenol-chloroform separation. DNA was precipitated from the aqueous phase using isopropanol. Protein was precipitated from the cytosolic fraction phenol phase using 0.1M ammonium acetate in methanol [53].

### qRT-PCR

BMDM were infected in 6-well plates. RNA was extracted at indicated time points using RNeasy Mini Kits (Qiagen), DNA was removed using RQ1 RNase-Free DNase (Promega), and cDNA was generated using Reverse Transcription System (Promega). IFNβ was normalized to β-actin (ng IFNβ/ng β-actin). DNA was quantified directly following cell fractionation and DNA precipitation. Cytosolic DNA was normalized to undisturbed cell DNA ((ng cytosolic DNA)/(ng total undisturbed cell DNA)*100). Amount in ng of each gene was determined using standards that were generated independently of experimental samples and that contained abundant levels of each gene. The sequences were as follows: IFNβ *fwd* CAGCTCCAAGAAAGGACGAAC, *rvs* GGCAGTGTAACTCTTCTGCAT; β-actin *fwd* AGTGTGACGTTGACATCCGTA, *rvs* GCCAGAGCAGTAATCTCCTTCT; D-loop *fwd* AATCTACCATCCTCCGTGAAACC, *rvs* TCAGTTTAGCTACCCCCCCAAGTTTAA; SigF *fwd* GCGGGTCGGGCTGGTCAAC, *rvs* CCTCGCCCATGATGGTAGGAAC; dnaA *fwd* CGACAACGACGAGATTGATGA, *rvs* CGGTAGCGGAATCGGTATTG.

### ELISA

BMDM were infected in 24-well plates. 48 hr post infection cell culture supernatants were collected from infected cells, filtered with 0.22 μm SpinX filters (Corning), and assayed for mouse IFNβ (PBL Interferon Source) and TNF (eBioscience).

### Fluorescence microscopy

BMDM were infected in Lab-Tek chamber slides. Cells were washed at indicated time points and fixed in 1% PFA overnight at 4°C. Primary antibodies FK2 (Millipore) and Galectin-3 (eBioscience) and secondary antibodies Alexa Fluor 488 goat anti-mouse and Alexa Fluor 488 chicken anti-rat (Invitrogen) were used at 1:1000. Slides were visualized on a Leica Leitz DMRB upright microscope. For superoxide analysis BMDM were incubated with 5 μM MitoSOX in DMEM for 25 min at 37°C, washed in warm PBS, and fixed in 1% PFA overnight at 4°C. Slides were visualized on a Zeiss LSM710 Multiphoton microscope. Mean cell fluorescence intensity was determined using ImageJ.

### LiveBLAzer FRET assay

BMDM were infected in black-wall, clear-bottom 96-well plates. 24 hr post infection CCF4-AM with 2.5 μM probenecid was added to cells at room temperature (LiveBLAzer FRET-B/G Loading Kit; Invitrogen). After 2 hr cells were washed 3x in PBS with 2.5 μM probenecid. Cells were fixed in 4% PFA for 30 min and then overnight in 1% PFA at 4°C. Slides were visualized on a Nikon Eclipse Ti inverted microscope and 450nm:520nm ratios of the area directly surrounding each bacterium were determined using NIS-Elements Imaging Software.

### Luminescent ATP detection

BMDM were grown for 6 days using 20 ng/mL M-CSF (PeproTech) and then were plated in 96-well plates in M-CSF media without glucose and with 5 mM galactose. Cells were grown for 24 hours in the presence of galactose and absence of glucose prior to infection and throughout infection. On day 7 BMDM were infected at the MOI reported in the text. 24 hr post infection intracellular ATP was measured using a Luminescent ATP Detection Assay Kit (Abcam).

### Immunoblotting

Protein from TN1 lysates or phenol extracts was denatured at 95°C for 5 min and quantified using Pierce BCA Protein Assay Kit. Samples were normalized to total amount of protein, separated by SDS-PAGE on 12% Tris-HCl gels (BioRad), and transferred onto 0.2 μm nitrocellulose membranes. Blots were incubated with anti-β-actin (Cell Signaling), anti-Complex Vα (Abcam), and anti-pyruvate dehydrogenase E1α (Abcam).

## Supporting Information

S1 Table Mycobacterial strains used in this study. (PDF)

S2 Table Differences in bacterial numbers and TNF do not explain differences in IFNβ induction between MTBC strains. ANCOVA models were run with IFNβ secretion at 48 hr post infection as the dependent variable, strain (1182, H37Rv, 4334) as a fixed factor, CFU collected at 3, 24 and 48 hr post infection as a covariate, and TNF secretion at 48 hr post infection as a covariate. No interactions were significant and therefore were removed from the models. Analysis was done in SPSS.
(PDF)

S1 Fig Access to the host cytosol does not vary by mycobacterial strain. A-B) BMDM were infected with the indicated dsRed-expressing bacterial strains at an MOI of 10, and then incubated with the LiveBLAzer FRET substrate at 24 hr post infection. Mtb in the cytosol cleaves the substrate and disrupts FRET; emission signals at 520 nm indicate no Mtb cytosolic access and emission signals at 450 nm indicate Mtb cytosolic access. A) Representative images are shown, with bacterial strains shown in white, the 450 nm emission shown in magenta, and the 520 nm emission shown in green. B) Three images were taken at 20x magnification for each well (3 wells per strain) and 450:520nm ratios were determined for the area directly surrounding each bacterium. Ratios were plotted on a histogram for each strain and fit to a Gaussian distribution using the linear regression function in Prism.
(TIF)

S2 Fig mtDNA in the cytosol is positively correlated with IFNβ induction during Mtb infection. A-C) BMDM were treated with MitoQ or control (dTPP) for 4 hours and then infected with the indicated bacterial strains at an MOI of 5. 24 hr post infection supernatants were collected for IFNβ quantification by ELISA and cells were fractionated. Amount of DNA in cytosolic fractions was determined using gene-specific primers for mitochondrial (A), nuclear (B), and bacterial (C) DNA; amount in ng was determined using standards that were generated independently of experimental samples and that contained abundant levels of each gene. Pearson correlation coefficient (r) of IFNβ induction (y-axis) and DNA in the cytosol (x-axis) is shown. Results shown are from the same experiment as those shown in Fig 5C–5H.
(TIF)
