# Supplementary material for: The Mechanism for Type I Interferon Induction by Mycobacterium tuberculosis is Bacterial Strain-Dependent
Source: PLoS Pathog. 2016 Aug 8;12(8):e1005809. doi: 10.1371/journal.ppat.1005809 (PMC4976988; doi:10.1371/journal.ppat.1005809)
Supplement: S1 Table — (PDF) [file ppat.1005809.s001.pdf]

Mtb strain	Lineage number	Lineage name	Family	Characteristics
H37Rv	4	Euro-America	Euro-America	Virulent strain derived from the parent strain H37 that was isolated from a 19-year old male with chronic pulmonary tuberculosis in New York[1].
H37Rv ΔEsx-1				Negative control strain. Does not secrete ESAT-6[2], access the cytosol[3], or induce IFNβ[4].
1182	6	West Africa	West Africa	Strain with reduced virulence in humans[5] and in mice[6] compared to Lineage 4 and Lineage 2 strains. Isolated from a male with pulmonary tuberculosis in The Gambia.
4334	2	East Asia	Beijing	Strain with increased virulence in guinea pigs compared to other Lineage 2, Beijing family strains[7]. Isolated from a patient with pulmonary tuberculosis in San Francisco.

[1]Steenken & Gardner, Am Rev Tuberc (1946); [2]Unpublished data; [3]Houben *et al*, Cell Microbiol (2012); Simeone *et al*. PLoS Pathog (2012); [4]Simeone *et al*. PLoS Pathog (2015), [5]de Jong *et al*. J Infect Dis (2008), [6]Bold *et al*. J Infect Dis (2012), [7]Kato-Maeda *et al*. Clin Vaccine Immunol (2012).
