# Supplementary material for: The Mechanism for Type I Interferon Induction by Mycobacterium tuberculosis is Bacterial Strain-Dependent
Source: PLoS Pathog. 2016 Aug 8;12(8):e1005809. doi: 10.1371/journal.ppat.1005809 (PMC4976988; doi:10.1371/journal.ppat.1005809)
Supplement: S2 Table — ANCOVA models were run with IFNβ secretion at 48 hr post infection as the dependent variable, strain (1182, H37Rv, 4334) as a fixed factor, CFU collected at 3, 24 and 48 hr post infection as a covariate, and TNF secretion at 48 hr post infection as a covariate. No interactions were significant and therefore were removed from the models. Analysis was done in SPSS. (PDF) [file ppat.1005809.s002.pdf]

	Effect	*F*	df	*P*
**MOI 1**	CFU	0.363	1	0.579
	TNF	0.084	1	0.787
	Strain	96.127	2	<0.001
**MOI 5**	CFU	1.636	1	0.27
	TNF	0.507	1	0.516
	Strain	35.137	2	0.003
**MOI 10**	CFU	0.385	1	0.569
	TNF	0.179	1	0.694
	Strain	9.254	2	0.032
