# Supplementary figures and images for: The Mechanism for Type I Interferon Induction by Mycobacterium tuberculosis is Bacterial Strain-Dependent
Source: PLoS Pathog. 2016 Aug 8;12(8):e1005809. doi: 10.1371/journal.ppat.1005809 (PMC4976988; doi:10.1371/journal.ppat.1005809)

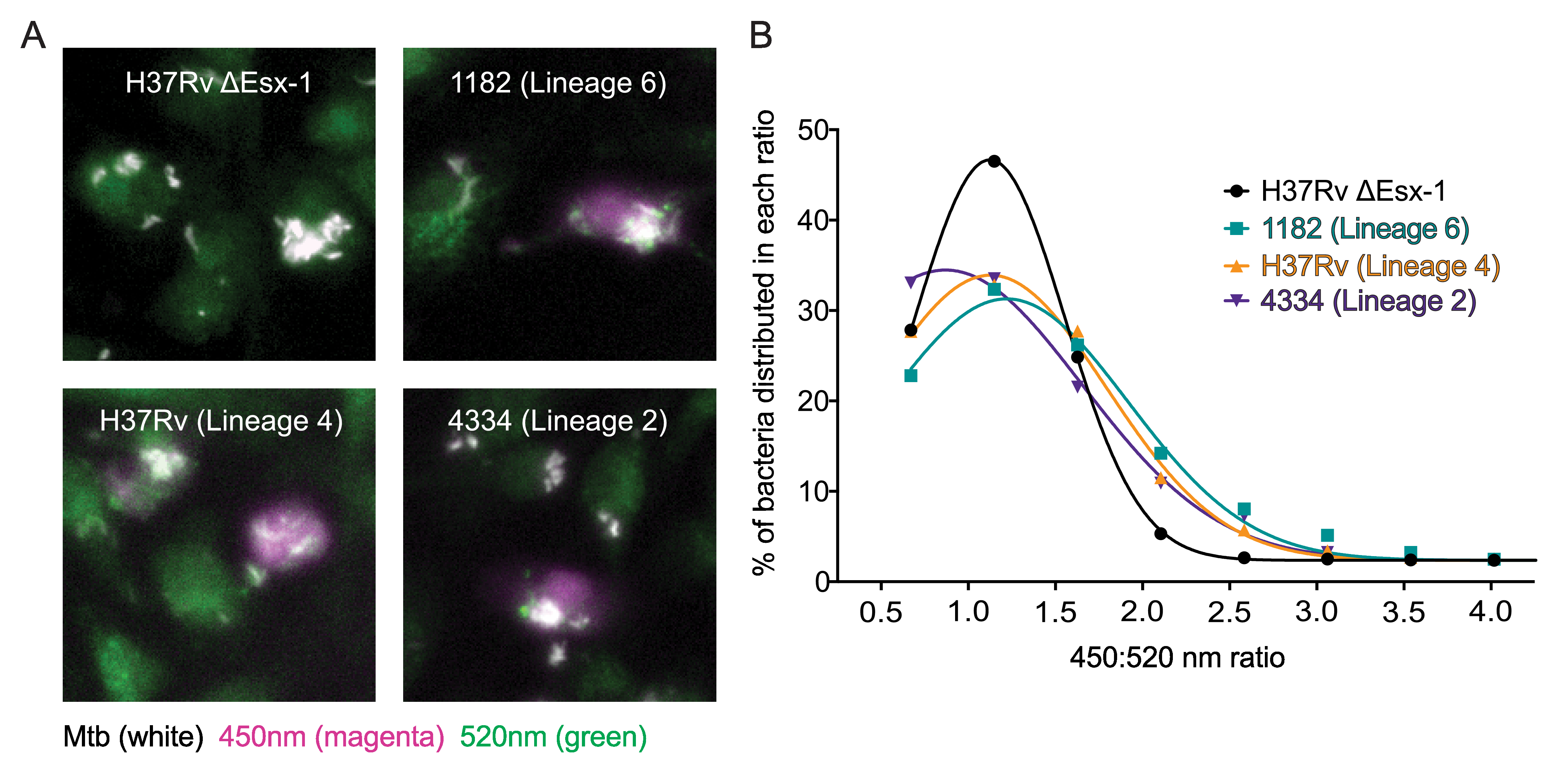

Supplement: S1 Fig — A-B) BMDM were infected with the indicated dsRed-expressing bacterial strains at an MOI of 10, and then incubated with the LiveBLAzer FRET substrate at 24 hr post infection. Mtb in the cytosol cleaves the substrate and disrupts FRET; emission signals at 520 nm indicate no Mtb cytosolic access and emission signals at 450 nm indicate Mtb cytosolic access. A) Representative images are shown, with bacterial strains shown in white, the 450 nm emission shown in magenta, and the 520 nm emission shown in green. B) Three images were taken at 20x magnification for each well (3 wells per strain) and 450:520nm ratios were determined for the area directly surrounding each bacterium. Ratios were plotted on a histogram for each strain and fit to a Gaussian distribution using the linear regression function in Prism. (TIF) [file ppat.1005809.s003.tif]

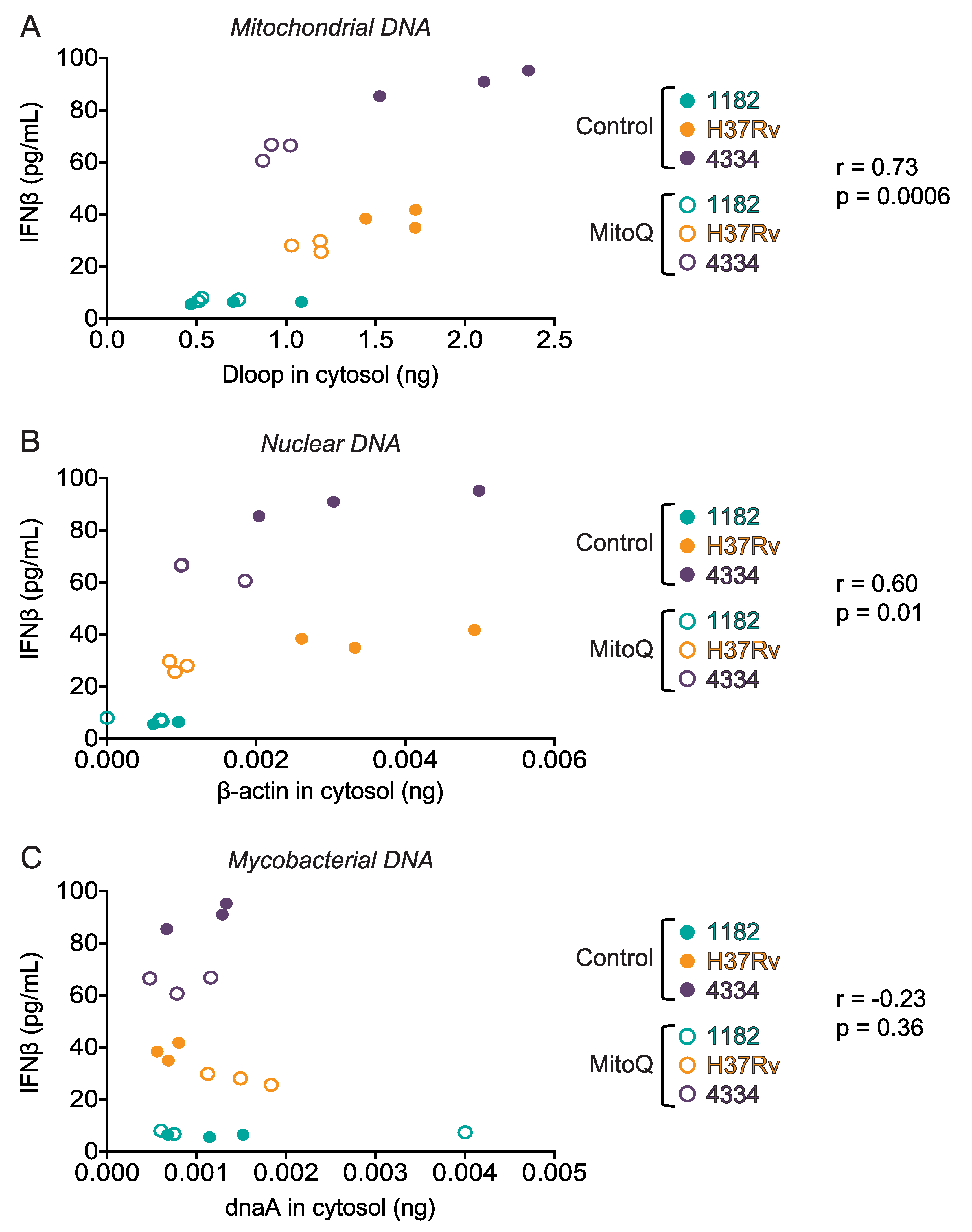

Supplement: S2 Fig — A-C) BMDM were treated with MitoQ or control (dTPP) for 4 hours and then infected with the indicated bacterial strains at an MOI of 5. 24 hr post infection supernatants were collected for IFNβ quantification by ELISA and cells were fractionated. Amount of DNA in cytosolic fractions was determined using gene-specific primers for mitochondrial (A), nuclear (B), and bacterial (C) DNA; amount in ng was determined using standards that were generated independently of experimental samples and that contained abundant levels of each gene. Pearson correlation coefficient (r) of IFNβ induction (y-axis) and DNA in the cytosol (x-axis) is shown. Results shown are from the same experiment as those shown in Fig 5C–5H. (TIF) [file ppat.1005809.s004.tif]
